# Preliminary Evidence for Adipocytokine Signals in Skeletal Muscle Glucose Uptake

**DOI:** 10.3389/fendo.2018.00295

**Published:** 2018-06-07

**Authors:** Akihiro Kudoh, Hiroaki Satoh, Hiroyuki Hirai, Tsuyoshi Watanabe, Michio Shimabukuro

**Affiliations:** ^1^Department of Diabetes, Endocrinology, and Metabolism, Fukushima Medical University, Fukushima-City, Japan; ^2^Department of Metabolism and Endocrinology, Juntendo University Graduate School of Medicine, Tokyo, Japan; ^3^Department of Internal Medicine, Fukushima Rosai Hospital, Iwaki, Japan

**Keywords:** adipose tissue, insulin resistance, adipocytokines, glucose transporter 4, skeletal muscle

## Abstract

The cross talk between the adipose tissue and insulin target tissues is a key mechanism for obesity-associated insulin resistance. However, the precise role of the interaction between the skeletal muscle and adipose tissue for insulin signaling and glucose uptake is questionable. L6 myocytes were co-cultured with or without 3T3-L1 adipocytes (~5 × 10^3^ cells/cm^2^) up to 24 h. Glucose uptake was evaluated by 2-[^3^H] deoxyglucose uptake assay. Levels of mRNA expression of *Glut1* and *Glut4* and mitochondrial enzymes were analyzed by quantitative real-time reverse transcription polymerase chain reaction. Levels of Glut1 and Glut4 protein and phosphorylation of Akt (Ser473 and Thr308) were analyzed by immunoblotting. Study 1: co-culture with 3T3-L1 adipocytes increased glucose uptake in dose- and time-dependent manner in L6 myocytes under insulin-untreated conditions. When co-cultured with 3T3-L1 cells, reactive oxygen species production and levels of *Glut1* mRNA and protein were increased in L6 cells, while these changes were abrogated and the glucose uptake partially inhibited by antioxidant treatment. Study 2: co-culture with 3T3-L1 adipocytes suppressed insulin-stimulated glucose uptake in L6 myocytes. Insulin-induced Akt phosphorylation at Ser473 decreased, which was proportional to 3T3-L1 density. Antioxidant treatment partially reversed this effect. Interactions between skeletal muscle and adipose tissues are important for glucose uptake under insulin-untreated or -treated condition through oxygen stress mechanism.

## Introduction

Obesity and the associated insulin resistance are thought to be the major cause of the global epidemic of type 2 diabetes. Overflow of lipids from the expanded adipose cells in obese individuals is considered as the key mechanism for the obesity-associated insulin resistance (1–3). In addition, other mechanisms related to the cross talk between the adipose tissue and insulin target tissues have been identified. The adipose tissue secretes an array of hormones and molecules called adipocytokines or adipokines. These molecules signal key organs to maintain metabolic homeostasis (4, 5). Several studies have shown the alteration in the secretory profile of adipocytokines in obese subjects and some of these factors are known to display important roles in inducing insulin resistance (6).

Roles of several adipocytokines on muscles insulin signaling have been identified (7). Dietze et al. reported a decrease in the phosphorylation of insulin receptor substrate-1 and AKT in skeletal muscle cells upon co-culture with human adipocytes and the subsequent inhibition of glucose transporter 4 (Glut4) translocation and glucose uptake (8, 9). By contrast, Vu et al. observed an increase in glucose uptake of L6 muscle cells co-cultured with 3T3-L1 adipocytes or incubated with adipocyte-conditioned media (10, 11). The discrepancy between two groups may be related to insulin present throughout co-culture versus insulin added only before metabolic assays (10).

Kozlovsky et al. reported that the reactive oxygen species (ROS) induced experimentally caused an increase in 2-deoxyglucose uptake in L6 myocytes and the increase was associated with the upregulated expression of *Glut1* mRNA and protein (12). Overabundance of ROS is mechanistically related with the multifactorial etiology of insulin resistance, primarily in the skeletal muscle tissue (13). We previously reported that ROS production from the accumulated fat leads to increased oxidative stress in blood and affects remote organs, including the skeletal muscle (14). Taken together, the interactions between the skeletal muscle and adipose tissue may alter insulin signaling and glucose uptake through increased ROS signals. However, this hypothesis needs to be elucidated.

In this study, we aimed to evaluate the interaction between the skeletal muscle and adipose tissue for insulin signaling and glucose uptake through ROS signals.

## Materials and Methods

### Materials

This research protocol had been approved by the institutional review board Fukushima Medical University.

3T3-L1 pre-adipocytes and L6 myocytes were purchased from the American Type Cell Collection (Manassas, VA, USA). Dulbecco’s modified Eagle’s medium (DMEM), streptomycin, trypsin, fetal bovine serum (FBS), TRIzol reagent, lithium dodecyl sulfate (LDS) sample buffer, and sample reducing agent were supplied by Invitrogen Life Technologies (Carlsbad, CA, USA). The cell culture insert was obtained from BD Falcon (Franklin Lakes, NJ, USA) and RNeasy kit, from QIAGEN Inc. (Valencia, CA, USA). The Akt antibody and anti-phospho-specific Akt (Ser473 and Thr308) was purchased from Cell Signaling Technology (Boston, MA, USA) and anti-glucose transporter Glut1 antibody, from Abcam (Burlingame, CA, USA).

Polyvinylidene difluoride (PVDF) transfer membranes were procured from Millipore Corp. (Bedford, MA, USA). iScript cDNA synthesis kit and iQ SYBR Green Supermix were provided by Bio-Rad Laboratories (Richmond, CA, USA). [^3^H] 2-deoxyglucose (2-DOG) was purchased from PerkinElmer Inc. (Waltham, MA, USA). The antioxidant Mn-TBAP chloride was supplied by Santa Cruz Biotechnology (Santa Cruz, CA, USA) and apocynin, by Abcam (Cambridge, UK). DCFDA (2′,7′-dichlorodihydrofluorescein diacetate) cellular ROS detection assay kit was obtained from Abcam (Burlingame, CA, USA).

### Cell Culture

Mouse 3T3-L1 pre-adipocytes and rat L6 myocytes were used in this study. 3T3-L1 cells were cultured and differentiated as previously described (15). 3T3-L1 fibroblasts were maintained in DMEM-high glucose (25 mM glucose, 1.8 mM CaCl_2_) medium containing 10% calf serum. Postconfluent fibroblasts were differentiated into adipocytes by adding 1 µg/mL insulin, 0.1 µg/mL dexamethasone, and 112 µg/mL isobutylmethylxanthine to the medium. L6 rat skeletal myoblasts were cultured in DMEM/F12 containing 10% FBS for 3 days and differentiation was induced by replacing the medium with the differentiation medium containing 2% horse serum.

### Co-Culture

We used 3T3-L1 pre-adipocytes for 14 days and L6 myocytes for 7 days after differentiation for co-culture experiments. The pre-adipocytes on cell culture inserts (pore size 1.0 µm) were washed with phosphate-buffered saline (PBS) and each cell culture insert plate was subsequently transferred to a culture plate containing differentiated and washed L6 myocytes. L6 cells were co-cultured with 3T3-L1 cells in serum-free DMEM/F12 supplemented with 0.1% bovine serum albumin (BSA). This resulted in an assembly, wherein the two cell types share the culture medium but remain separated. 2-DOG uptake assay, reverse transcription polymerase chain reaction (RT-PCR), and immunoblotting analysis were performed in six-well cell culture inserts, while ROS detection assay was carried out in 24-well cell culture inserts. Co-culture was conducted for 24 h.

### 2-Deoxyglucose Uptake Assay

Glucose uptake was initiated as previously described (15), with minor modifications. The differentiated 3T3-L1 adipocytes and L6 myocytes were co-cultured for 24 h. L6 cells were serum-starved for 6 h in KRP-HEPES buffer [10 mM HEPES pH 7.4, 131.2 mM sodium chloride (NaCl), 4.7 mM potassium chloride (KCl), 1.2 mM magnesium sulfate (MgSO_4_), 2.5 mM calcium chloride (CaCl_2_), and 2.5 mM monosodium phosphate (NaH_2_PO_4_)] for 30 min at 37°C. In case of insulin stimulation, we used 100 ng/mL concentration of insulin. Glucose uptake was determined in triplicates at each point of addition of 2-DOG ([^3^H] 0.1 μCi at a final concentration of 0.1 mM) in the KRP-HEPES buffer for 5 min at 37°C. Cells were washed thrice with ice-cold PBS and suspended in 1N sodium hydroxide (NaOH). All samples were subjected to quantification of radioactivity using LSC-6100 liquid scintillation counter (Aloka, Tokyo, Japan).

### Quantitative Real-Time Reverse Transcription Polymerase Chain Reaction Analysis

Total RNA was extracted from the cells using TRIzol reagent and purified using the RNeasy kit with RNase-free DNase I, according to the manufacturer’s instructions. We measured RNA quantity and quality by Gene Quant pro RNA/DNA calculator (Pharmacia biotech). Total RNA (1 µg) was reverse transcribed with the iScript cDNA synthesis kit according to the manufacturer’s instructions (Bio-Rad). The gene expression levels were measured by real-time quantitative PCR using a Bio-Rad iQ™5 real-time PCR detector system. We used iQ™5 Optical System software to calculate the relative gene expression. The forward (fwd) and reverse (rev) primer sequences are as follows: rGlut1 (GeneBank accession number 38197584; fwd: 5′-CCGCTTCCTGCTCATCAATC-3′; rev: 5′-CGACCCTCTTCTTTCATCTCC-3′), rGlut4 (GeneBank accession number 55250154; fwd: 5′-CAGTATGTTGCGGATGCTATG-3′; rev: 5′-TTAGGAAGGTGAAGATGAAGAAG-3′), rUcp3 (GeneBank accession number 47940721; fwd: 5′-CTGTAATGTGTCTGCCAAGTG-3′; rev: 5′-TGCTCTATCCTCCAGTGTCC-3′), rCPH (Cyclophilin A) (housekeeping gene) (GeneBank accession number 60688168; fwd: 5′-CACCACATGCTTGCCATCC-3′; rev: 5′-CTCCTTTGAGCTGTTTGCAG-3′). We selected commercially available Cyclophilin A primers from the Housekeeping Gene Primer Set (Takara Bio, Japan), which had been assessed for the stability.

### Immunoblotting Analysis

Serum-starved L6 cells were stimulated with 100 ng/mL insulin at 37°C for various time points, as indicated in each experiment. The cells were lysed in a solubilization buffer containing 20 mM Tris, 1 mM ethylenediaminetetraacetic acid, 140 mM NaCl, 1% Nonidet P-40, 50 U of aprotinin/mL, 1 mM sodium orthovanadate (Na_3_VO_4_), 1 mM phenylmethylsulfonyl fluoride, and 10 mM sodium fluoride (NaF) (pH 7.5) for 30 min at 4°C. The cell lysates were centrifuged at 15,000 rpm for 30 min at 4°C to remove insoluble material. For immunoblot analysis, the whole cell lysates (10 µg protein per lane) were denatured by boiling in LDS sample buffer and sample reducing agent. The gels were transferred to PVDF membranes using a semi-dry trans-blot apparatus (Bio-Rad). For immunoblotting, the membranes were probed with the relevant primary antibodies, followed by their incubation with horseradish peroxidase-conjugated secondary antibodies and chemiluminescence detection (Pierce, Rockford, IL, USA). For re-probing, the membranes were striped with a buffer (2% SDS, 100 mM 2ME, 62.5 mM Tris, pH 6.8) for 30 min at 50°C. The band intensities were quantified by densitometry using the Image-J software (NIH, USA).

### ROS Detection

We co-cultured L6 and 3T3-L1 on 24-well cell culture inserts (pore size 1.0 µm). The adipocytes were washed with PBS and each cell culture insert plate was subsequently transferred to the culture plates containing differentiated and PBS-washed L6 myocytes. L6 and 3T3-L1 cells were co-cultured in serum-free DMEM/F12 supplemented with 0.1% BSA with or without the antioxidants Mn-TBAP and apocynin for 24 h. We washed cells once in the optional buffer and stained them with DCFDA for 45 min. Cells were washed again in the optional buffer and the intensity of signal analyzed at maximum excitation and emission spectra of 485 and 535 nm, respectively, using Varioskan™ Flash (Thermo Scientific, Waltham, MA, USA). The change in the fluorescence intensity was reported in percentage as compared with control cells.

### Statistical Analysis

All data are presented as mean ± SD. One-way ANOVA followed by *post hoc* Tukey HSD test was used in statistical analyses unless otherwise indicated. Two groups comparison was made by unpaired two-tailed *t*-test. All analyses were performed using SPSS software (version 24.0; SPSS, Chicago, IL, USA). A value of *P* < 0.05 was considered to be statistically significant.

## Results

### Study 1: Insulin-Independent Glucose Uptake in L6 Myocytes Co-Cultured With 3T3-L1 Adipocytes

#### Time- and Density-Dependent Effects of 3T3-L1 Co-Culture

**Figure 1:**
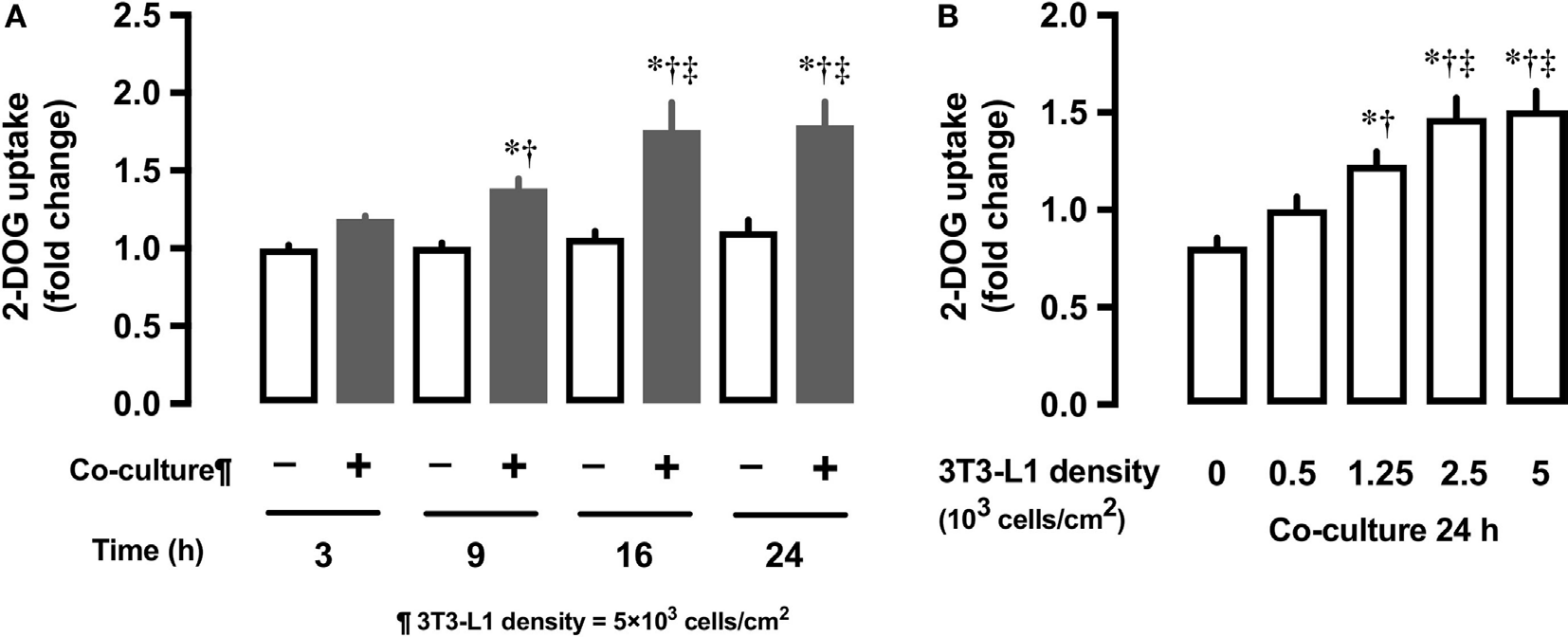
Effect of co-culture of L6 cells with 3T3-Ll on [^3^H]2-deoxyglucose (2-DOG) uptake of L6 cells. **(A)** Uptake of 2-deoxyglucose by L6 cells was measured in the absence (−, white bars) or presence (+, black bars) of adipocyte co-culture for 3–24 h. Values are mean ± SD (*n* = 5). *P* values by one-way ANOVA followed by post-hoc Tukey HSO test are shown. ANOVA *P* < 0.01, *: vs co-culture (−) 3 h, †: vs co-culture (+) 3 h, ‡: vs co-culture (+) 9 h. **(B)** Uptake of 2-deoxyglucose by L6 cells was measured in the presence of 3T3-Ll adipocyte co-culture for at indicated densities. Values are mean ± SD (*n* = 5). ANOVA *P* < 0.01, *: vs 3T3-Ll density 0, †: vs 3T3-L I density 0.5, ‡: vs 3T3-Ll density 1.25.

We first examined whether the co-culture with 3T3-L1 adipocytes affect the glucose uptake in L6 myocytes. In comparison with L6 cells grown in the absence of 3T3-L1 for 3 h, L6 cells co-cultured with 3T3-L1 showed up to 80% increase in 2-DOG uptake from 3 to 24 h (Figure 1A). At 24 h of co-culture, the level of 2-DOG uptake in L6 cells increased proportionally with the density of 3T3-L1 cells (Figure 1B).

#### Effects of 3T3-L1 Co-Culture on mRNA Expression of *Glut1* and *Glut4*

**Figure 2:**
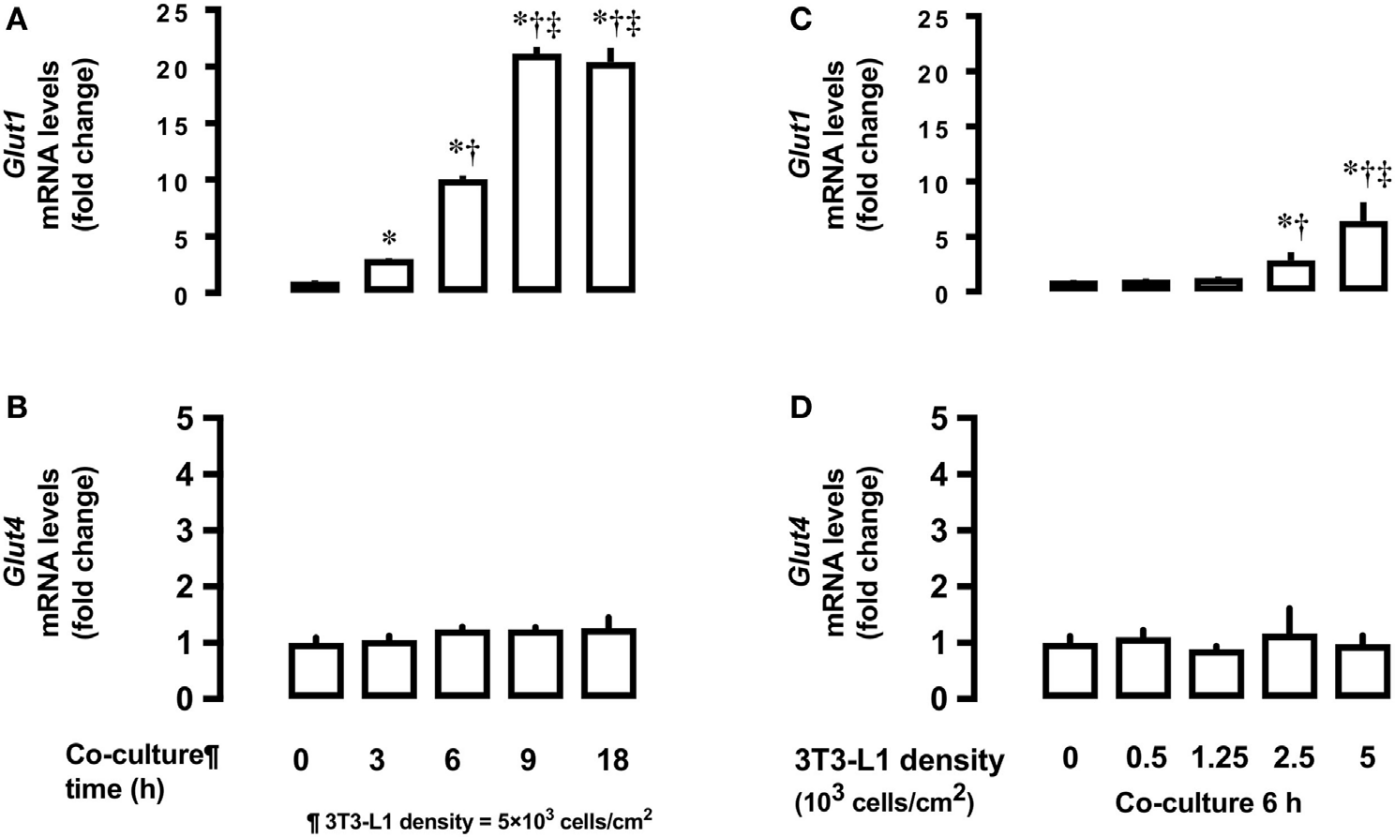
Effects of co-culture time and 3T3-Ll cell density on mRNA levels of *Glutl* and *Glut4* in L6 cells. L6 cells were incubated with 3T3-Ll adipocytes at various times at 5 × 10^3^ density and mRNA levels of **(A)** *Glut1* and **(B)** *Glut4* measured. L6 cells were incubated with 3T3-Ll adipocytes for 6 h at indicated densities and the mRNA levels of *Glutl*. *P* values by one-way ANOVA followed by post-hoc Tukey HSO test are shown. ANOVA *P* < 0.01, *: vs 0 h, †: vs 3 h, ‡: vs 6 h. **(C)** and *Glut4* **(D)** measured. Values are mean ± SD (*n* = 5). ANOVA *P* < 0.01, *: vs 3T3-LI density 0, †: vs 3T3-Ll density 1.25, ‡: vs 3T3-Ll density 2.5. Glutl, glucose transporter type I; Glut4, glucose transporter type 4.

Next, we measured time- and dose-dependent effects of 3T3-L1 on the mRNA expression of *Glut1* and *Glut4* in the basal state of L6 myocytes. Total RNA extracted from L6 cells co-cultured for 18 h with 5 × 10^3^ 3T3-L1 cells (Figures 2A,C) was used for mRNA quantification of *Glut1* and *Glut4*. Co-culture of L6 cells with 3T3-L1 cells resulted in an increase in the expression of *Glut1* mRNA in a time-dependent manner (20.1 times at 18 versus 0 h, *P* < 0.01, Figure 2A). The increase in *Glut1* mRNA expression was proportional to the density of 3T3-L1 cells (6.4 times at 5 × 10^3^ cells/cm^2^ versus 0 density, *P* < 0.01, Figure 2C). However, no change in the expression of *Glut4* mRNA was observed after co-culture with 3T3-L1 (Figures 2B,D).

#### Effects of 3T3-L1 Co-Culture on ROS Production

**Figure 3:**
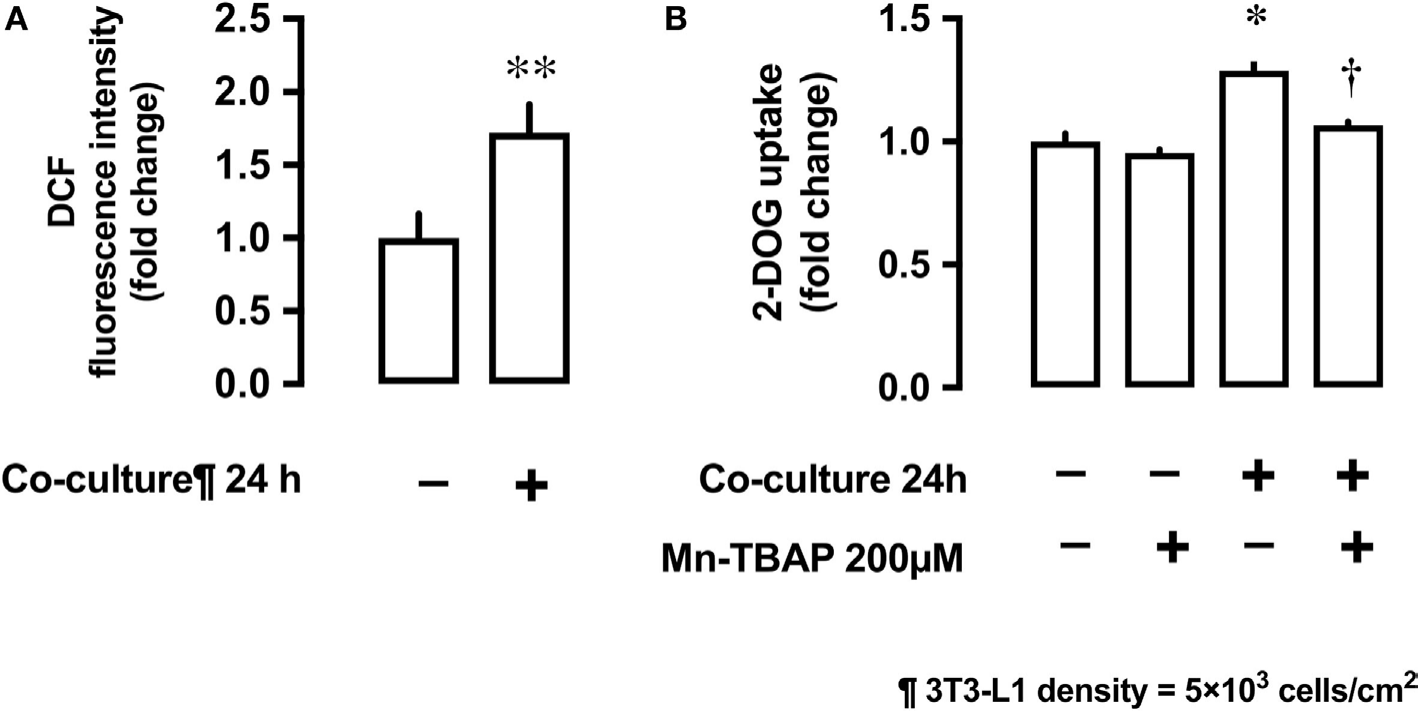
Effects of co-culture with 3T3-Ll cells on reactive oxygen species (ROS) production and [^3^H]2-deoxyglucose (2-DOG) uptake in L6 cells. **(A)** L6 cells were incubated for 24 h in co-culture with 3T3-Ll cells and the cellular ROS level detected by OCFOA intensity. Values are mean ± SD (*n* = 6) **P < 0.01 vs co-culture (−) by unpaired two-tailed t test. **(B)** L6 cells were incubated for 24 h in the absence or presence of 3T3-Ll cells and treated with the antioxidant Mn-TBAP (200 µM) for 24 h. Values are mean ± SD (*n* = 3). *P* values by one-way ANOVA followed by post-hoc Tukey HSO test are shown. ANOVA *P* < 0.01, *: vs co-culture (−), Mn-TBAP (−), †: vs co-culture (+), Mn-TBAP (−); DCF, dichlorofluorescein; 2-DOG, [^3^H]2-deoxyglucose.

Co-culture of L6 cells with 3T3-L1 resulted in an increase in the production of ROS by L6 cells. The ROS production was up to 1.7-fold higher in presence of co-culture as compared with the control (Figure 3A). To evaluate the involvement of ROS in glucose uptake, we incubated L6 cells with 3T3-L1 cells with or without the antioxidant Mn-TBAP. The stimulating effect of 3T3-L1 co-culture on the glucose uptake was abrogated in the presence of Mn-TBAP (Figure 3B).

#### Effects of ROS and Antioxidants on mRNA and Protein Levels of Glut1

**Figure 4:**
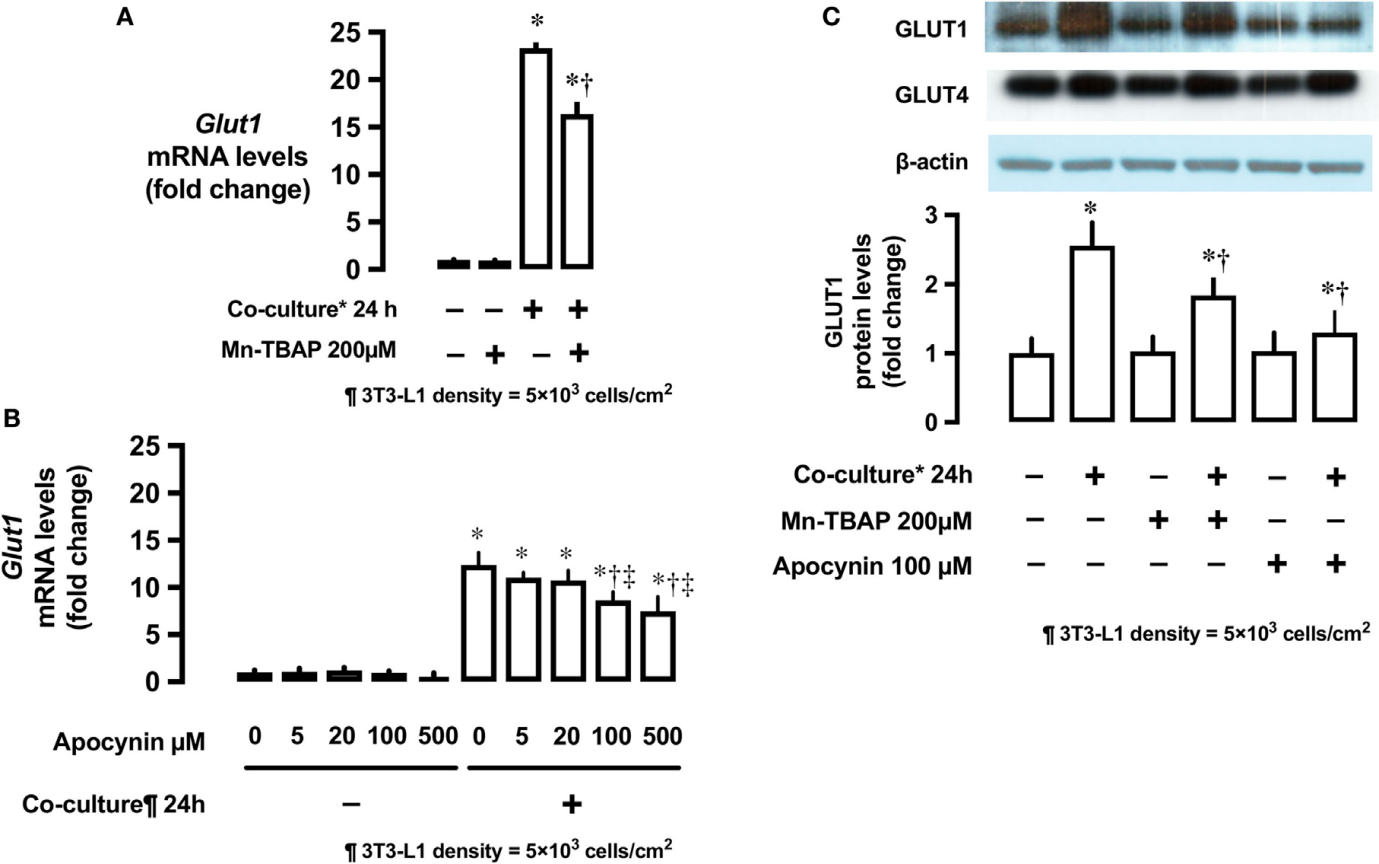
Effects of co-culture with 3T3-Ll cells and antioxidants on mRNA and protein levels of Glut1. **(A)** L6 cells were incubated with 3T3-Ll adipocytes or antioxidant Mn-TBAP (200 µM) for 24 h. mRNA levels of Glut1 were measured by quantitative real-time RT-PCR. Values are mean ± SD (*n* = 5). *P* values by one-way ANOVA followed by post-hoc Tukey HSO test are shown. ANOVA *P* < 0.01, *: vs co-culture (−), Mn-TBAP (−), †: vs co-culture (+), Mn-TBAP (−). **(B)** L6 cells were incubated with 3T3-Ll adipocytes or antioxidant apocynin at indicated concentrations for 24 h. mRNA levels of Glut1 were measured by quantitative realtime RT-PCR. Values are mean ± SD (*n* = 5). ANOVA *P* < 0.01, *: vs co-culture (−), apocynin 0 µM, †: vs co-culture (+), Apocynin 0 µM, ‡: vs co-culture (+), Apocynin 20 µM, **(C)** L6 cells were incubated with 3T3-LJ adipocytes, Mn-TBAP, or apocynin for 24 h. Levels of Glutl protein were measured by immunoblotting. Values are mean ± SD (*n* = 5). ANOVA *P* < 0.01, *: vs co-culture (−), Mn-TBAP (−), Apocynin (−), †: co-culture (+), Mn-TBAP (−), Apocynin (−), Glutl, glucose transporter type 1.

The antioxidant Mn-TBAP partially inhibited the stimulatory effects of 3T3-L1 cells on *Glut1* mRNA expression by around 40% (Figure 4A). In addition, NADPH oxidase inhibitor, apocynin, also suppressed the effects of 3T3-L1 cells on *Glut1* mRNA expression in a dose-dependent manner (Figure 4B). No change in the protein levels of Glut4 was observed; however, Glut1 protein level increased in the co-culture system (Figure 4C). The increase in Glut1 level was significantly inhibited by Mn-TBAP and apocynin (Figure 4C).

#### Effects of 3T3-L1 Co-Culture on mRNA Expression of the Potent Regulators of Mitochondrial Biogenesis *Pgc1*α and *Ucp3*

**Figure 5:**
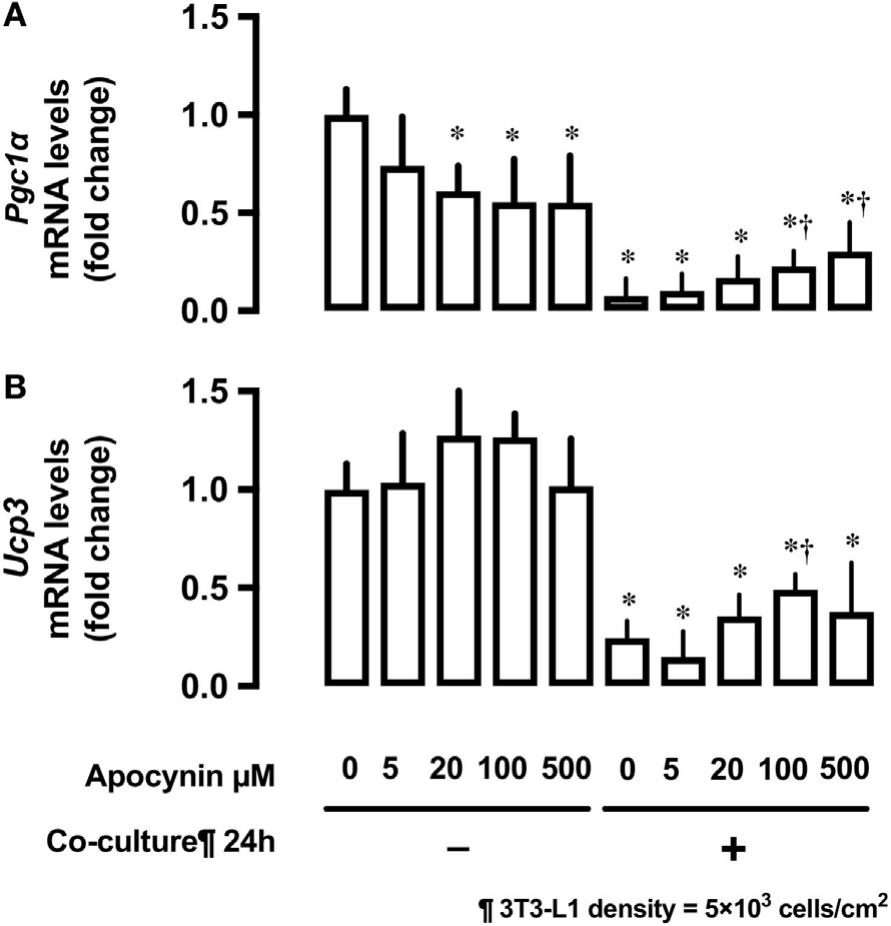
Effects of co-culture with 3T3-Ll cells and antioxidants on mRNA levels of *Pgc1*α and *Ucp3*. L6 cells were incubated with 3T3-Ll adipocytes or antioxidant apocynin at indicated concentrations for 24 h. mRNA levels of **(A)***Pgc1*α and **(B)***Ucp3* were measured by quantitative real-time RT-PCR. Values are mean ± SD (*n* = 3). *P* values by one-way ANOVA followed by post-hoc Tukey HSO test are shown. ANOVA *P* < 0.01, *: vs co-culture −, Apocynin 0 µM, †: vs co-culture (+), Apocynin 0 µM, PGC1α, peroxisome proliferator-activated receptor (PPAR)-γ coactivator 1α; Ucp3, uncoupling protein 3.

Co-culture of L6 cells with 3T3-L1 cells strongly suppressed the mRNA expression of proliferator-activated receptor γ coactivator 1-α (*Pgc1*α) and uncoupling protein 3 (*Ucp3*) in L6 myocytes (Figures 5A,B). Apocynin partially restored the inhibitory effects of 3T3-L1 on mRNA expression of *Pgc1*α and *Ucp3*.

### Study 2: Insulin-Dependent Glucose Uptake in L6 Myocytes Co-Cultured With 3T3-L1 Adipocytes

#### Effects of 3T3-L1 Co-Culture on Insulin-Stimulated 2-DOG Uptake

**Figure 6:**
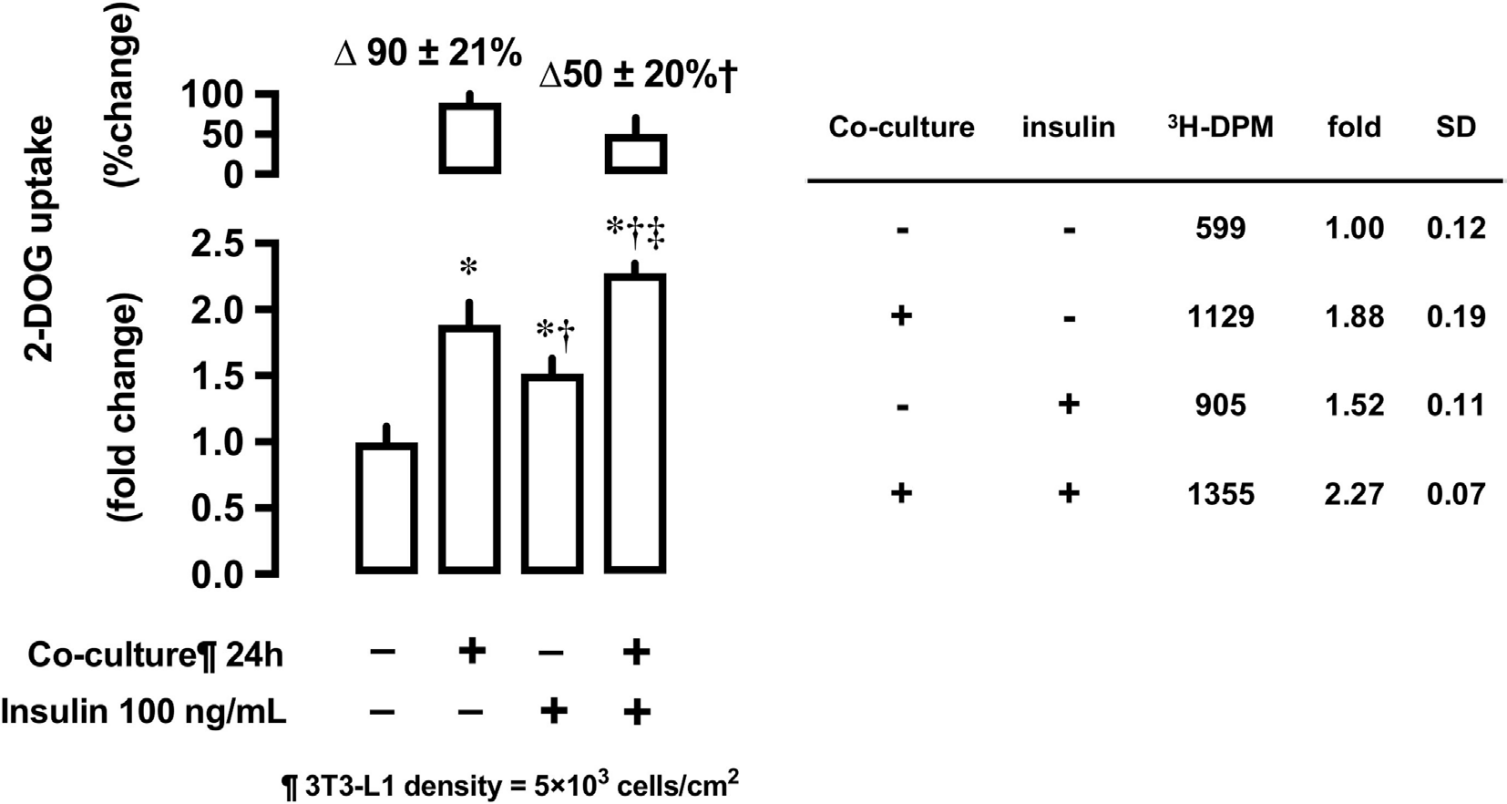
Effects of co-culture with 3T3-Ll cells on insulin-stimulated [^3^H]2-deoxyglucose (2-DOG) uptake in L6 cells. Uptake of 2-deoxyglucose in L6 cells was measured in the absence (−) or presence (+) of 3T3-LI adipocyte co-culture for 24 h. Serum-starved L6 cells were treated without or with insulin (100 ng/mL) for 30 min. Values are mean ± SD (*n* = 5) of percent change to reference (co-culture [−]) and fold change to reference (co-culture [−] and insulin [−]). *P* values by one-way ANOVA followed by post-hoc Tukey HSO test are shown. ANOVA *P* < 0.01 *: vs co-culture (−), insulin (−), †: vs co-culture (+), insulin (−), ‡: vs co-culture (−), insulin (+).

After 24-h co-culture with 3T3-L1 cells, L6 cells were serum starved and treated with or without insulin at 100 ng/mL concentration for 30 min and their 2-DOG uptake measured. Co-culture with 3T3-L1 increased 2-DOG uptake by 90% in insulin-untreated cells, while the increase in 2-DOG uptake was limited to 50% in insulin-treated cells (*P* < 0.05) (Figure 6).

#### Effects of 3T3-L1 Co-Culture on the Phosphorylation of Akt (Ser473 and Thr308)

**Figure 7:**
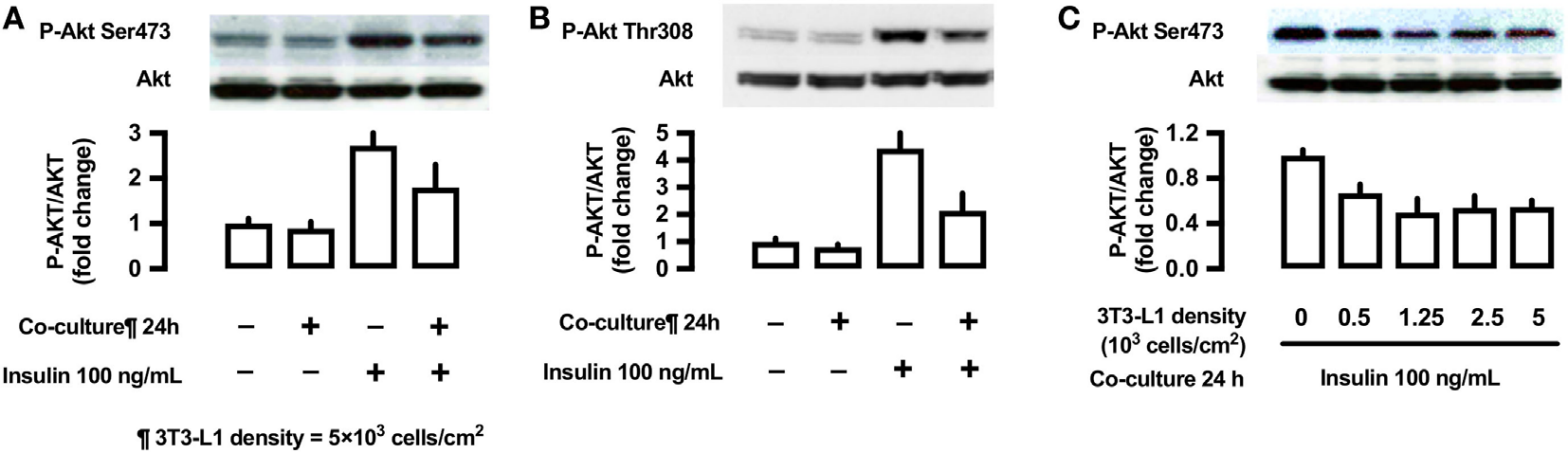
Effects of co-culture with 3T3-Ll cells on insulin-stimulated phosphorylation of Akt (Ser473 and Thr308) in L6 cells. **(A,B)** L6 cells were incubated for 24 h in coculture with 3T3-Ll cells at a density 5 × 10^3^ cells/cm^2^ and stimulated with insulin (100 ng/mL) for 10 min. Following whole cell lysis, the protein samples were immunoblotted with antiphospho Akt (Ser473 and Thr308) antibody. *P* values by one-way ANOVA followed by post-hoc Tukey HSD test are shown. ANOVA *P* < 0.01, *: vs co-culture (−), insulin (−), †: vs co-culture(−), insulin (+). **(C)** L6 cells were incubated for 24 h in co-culture with 3T3-Ll cells at indicated densities and stimulated with insulin (100 ng/mL) for 10 min. Following whole cell lysis, the protein samples were immunoblotted with anti-phospho-Akt (Ser473) antibody. ANOVA *P* < 0.01, *: vs 3T3-L1 density 0. Values are mean ± SD; *n* = 4 **(A)**, *n* = 3 **(B)**, and *n* = 3 **(C)**.

To evaluate the effects of 3T3-L1 co-culture on Akt, L6 cells were co-cultured with 3T3-L1 cells for 24 h, followed by their stimulation with insulin (100 ng/mL) for 10 min. The cells were lysed and immunoblotted using anti-phospho-Akt (Ser473 and Thr308) antibodies. Insulin stimulation markedly increases Akt phosphorylation at both sites (lane 3, Figures 7A,B); however, these effects were partially inhibited by 3T3-L1 co-culture (lane 4, Figures 7A,B). The decrease in insulin-induced Akt phosphorylation at Ser473 was proportional to the density of 3T3-L1 (Figure 7C).

### Antioxidant Apocynin and Insulin-Stimulated Phosphorylation of Akt (Ser473 and Thr308)

**Figure 8:**
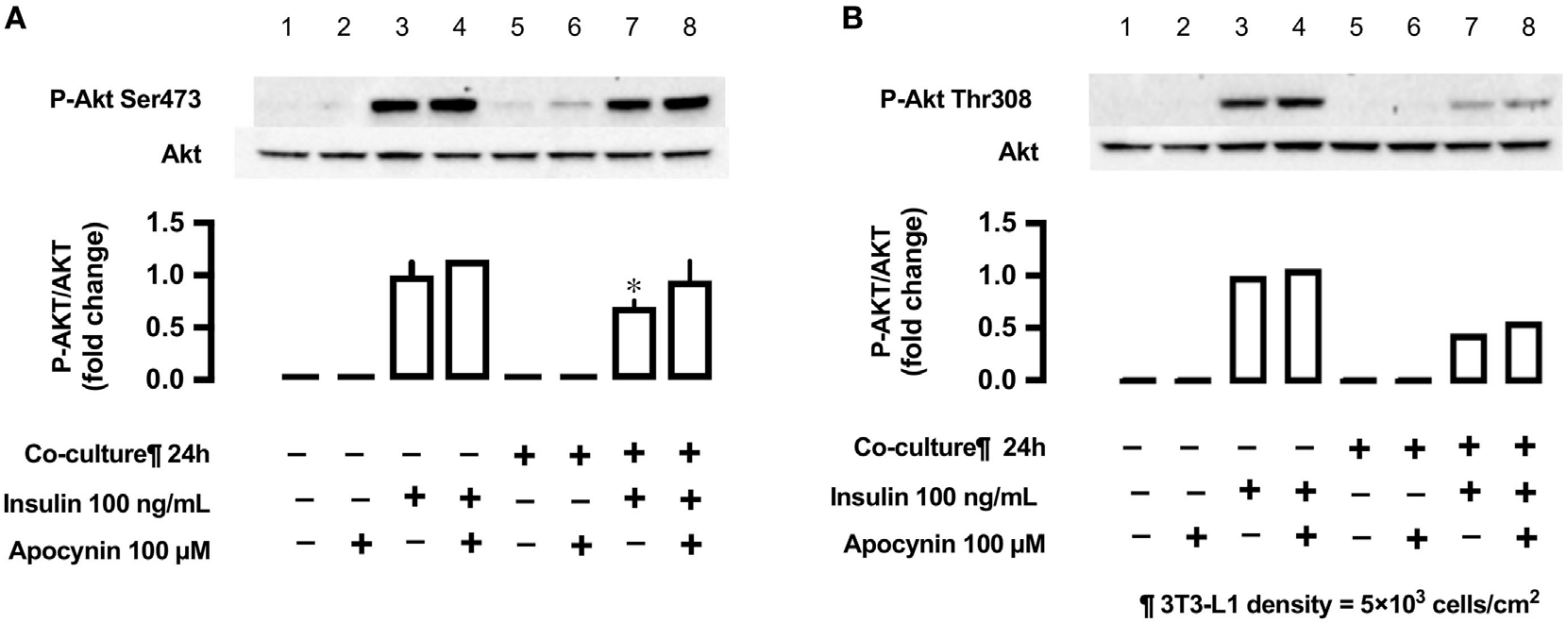
Effects of co-culture with 3T3-LI cells and antioxidant apocynin on insulin-stimulated phosphorylation of Akt [**(A)** Ser473 and **(B)** Tbr308] in L6 cells. L6 cells were incubated without or with apocynin (100 µM) for 24 h in absence or presence of 3T3-Ll cells co-culture at a density 5 × 10^3^ cells/cm^2^ and stimulated with or without insulin (100 ng/mL) for 10 min. Following whole cell lysis, the protein samples were immunoblotted with anti-phospho Akt (Ser473 and Thr308) antibody. **(A)** Values are mean ± SD (*n* = 3). *P* values by one-way ANOVA followed by post-hoc Tukey HSD test are shown. ANOVA *P* < 0.01, *: vs co-culture (−), insulin (+), apocynin (+). **(B)** *n* = 1.

In L6 cells, insulin-stimulated phosphorylation of Akt (Ser473) was decreased in L6 cells un-co-cultured versus co-cultured with 3T3-L1 cells (lane 3 versus lane 7; Figure 8), but the antioxidant apocynin partially restored the decrease in Akt Ser473 phosphorylation (lane 8; Figure 8). For reference, we provided (*n* = 1) experiment for Akt Thr308.

## Discussion

The present study highlights two findings. First, co-culture of L6 myocytes with 3T3-L1 adipocytes resulted in an increase in the glucose uptake in a dose- and time-dependent manner in L6 myocytes in the absence of insulin stimulation. In addition, co-culture treatment increased ROS production and *Glut1* mRNA and protein level in L6 cells. The addition of antioxidants resulted in the partial inhibition of ROS production and Glut1 expression as well as glucose uptake. Thus, stimulation of glucose uptake may be induced in the skeletal muscle by adipocytes *via* ROS production. Second, co-culture of L6 myocytes with 3T3-L1 adipocytes resulted in the suppression of insulin-stimulated glucose uptake in L6 myocytes. In addition, 3T3-L1 co-culture markedly inhibited the increase in Akt phosphorylation at both sites. The decrease in insulin-induced Akt phosphorylation at Ser473 was proportional to the density of 3T3-L1 cells, and addition of the antioxidant could partially recover Akt phosphorylation, suggestive of the association between ROS production by co-culture and the decreased glucose uptake and Akt signal in insulin-stimulated cells. Taken together, the current study demonstrates that the interaction between the skeletal muscle and adipose tissue resulted in alterations in glucose uptake by skeletal muscle cells with or without insulin stimulation and these changes are, at least in part mediated, through ROS signaling.

### Insulin-Independent Glucose Uptake in L6 Cells Co-Cultured With 3T3-L1 Cells

Co-culture of L6 cells with 3T3-L1 adipocytes resulted in a dose- and time-dependent increase in the glucose uptake in L6 cells, which reached up to ~180% (Figure 1). Glut1 transporter is thought to be predominantly involved in basal glucose uptake (16), while Glut4 plays a major role in mediating insulin-stimulated glucose uptake in muscle cells (17). Under co-culture conditions, the level of *Glut1* mRNA and protein increased in the insulin-untreated L6 cells, while no change in the level of Glut4 was observed (Figure 2). The increased glucose uptake in the insulin-untreated cells may be explained by the upregulation in the expression of Glut1 but not Glut4, as observed in previous studies (16, 17). As the enhanced expression of *Glut1* mRNA and protein was reported to be associated with ROS production (12), we evaluated whether ROS production in L6 cells was increased under co-culture condition and if ROS upregulates mRNA and protein level of Glut1. We used two potent antioxidants: Mn-TBAP, which has catalytic activities similar to those of ROS-scavenging enzymes superoxide dismutase and catalase and protects cells from hydrogen peroxide (H_2_O_2_) damage (18), and apocynin—the most selective inhibitor of NADPH oxidases that prevents translocation of p47phox from the cytosol to Nox1/2 catalytic membrane domain (19). The increase in the mRNA and protein level of Glut1 was abrogated and 2-DOG was partially suppressed following addition of antioxidants, supporting the hypothesis that the association between ROS and Glut1 expression was attributable to glucose uptake under insulin-untreated condition. In the present study, adipocyte-derived trigger(s) for ROS production was not evaluated. We and others have shown that free fatty acids (14) or other proinflammatory adipocytokines (20–22) such as tumor necrosis factor alpha, interleukin 1, monocyte chemotactic protein 1, and resistin may trigger ROS production through the activation of NADPH oxidase or other inflammatory mechanisms. These factors may have played role in the interaction between the muscle cells and adipocytes observed in our study.

Mitochondrial metabolism is responsible for majority of the ROS production in various cells (23). PGC1α is a potent regulator of energy metabolism and mitochondrial biogenesis (24, 25) and particularly controls the expression of uncoupling protein (Ucp) 2 and Ucp3, both of which are now understood to be important regulators of ROS formation (26). Ucp3 plays a role in the protection of mitochondria against lipid-induced oxidative damage either by facilitating mitochondrial fatty acid export or by adjusting ROS production (26). Sun and Zemel reported that 3T3-L1 adipocyte-conditioned medium reduced mitochondrial abundance and *Ucp3* expression in C2C12 myocytes (27). In agreement with this report, we observed that the expression of *Pgc1*α and *Ucp3* mRNA was downregulated under 3T3-L1 co-culture condition. Apocynin treatment partially restored *Pgc1*α and *Ucp3* mRNA expression, suggesting that the oxidative stress caused by adipocytes suppressed the mitochondrial function in skeletal muscle cells. From our study results, we are not able to conclude whether UCP3 regulates ROS, or ROS regulate UCP3, or both.

Thus, stimulation with adipocyte-derived molecules such as free fatty acids or adipocytokines may result in increased ROS production, which in turn upregulates the expression of *Glut1* mRNA and protein and increases glucose uptake in insulin-untreated cells.

### Insulin-Dependent Glucose Uptake in L6 Cells Co-Cultured With 3T3-L1 Cells

In contrast to the insulin-untreated condition, co-culture of L6 cells with 3T3-L1 adipocytes resulted in the suppression of insulin-stimulated glucose uptake. In L6 cells co-cultured with 3T3-L1 adipocytes, glucose uptake increased under insulin-untreated condition but decreased upon insulin treatment. This contrasting observation may be associated with “insulin present throughout co-culture versus insulin added only before metabolic assays,” as previously reported (10).

We further evaluated the effects of 3T3-L1 co-culture by measuring insulin signaling. The serine/threonine kinase Akt plays a crucial role in insulin-stimulated glucose transport (28). Our results demonstrate a decrease in the phosphorylation of Akt (Ser473) in L6 muscle cells under co-culture condition, while the addition of antioxidant almost completely restored the phosphorylation of Akt. Seyoum et al. reported that co-culture of 3T3-L1 cells with L6 cells increased IL-6 expression and decreased insulin-stimulated Akt phosphorylation (29). It is therefore suggested that the adipocytokine-induced ROS production is related to the decrease in insulin-stimulated Akt phosphorylation. Increased ROS production in L6 cells may be associated with several adipocytokines such as IL-6, TNF-α (8, 30), resistin (31), free fatty acid (32), angiotensin II (33), and visfatin (34). Although ROS-producing effects of each adipocytokine may be variable, synergism among multiple adipocytokines may lead to ROS production and decrease in insulin-dependent signals in skeletal muscle cells (7). In the current study, we cannot exclude that possibility that any changes in the Glut1 and Glut4 mRNA and protein levels in L6 cells might affect the insulin-treated glucose uptake. Other possible mechanism for adipocyte-derived molecules (adipocytokines)-induced glucose uptake can be Glut4-mediated one or other metabolic derangements. Dietze-Schroeder et al. reported that human skeletal muscle cells co-cultured with adipocyte-conditioned medium showed an impaired in response to insulin as well a reduction of Glut4 translocation (9). Suborganellar localization of Glut4 and/or metabolic derangements, both not mutually exclusive, might be involved.

This study has limitations. The suitability of 3T3-L1 and L6 cell lines needs to be taken into account. Both models have limitations as compared with freshly prepared adipocytes or muscle cells (23). Generation of 3T3-L1 adipocytes from pre-adipocytes requires at least 2 weeks. Furthermore, over-propagation or extensive passaging of 3T3-L1 decreases their ability to differentiate robustly into adipocytes. We used cell lines of two different species (mouse 3T3-L1 and rat L6). As the amino acid sequence for adipocytokines is preserved (strong homology observed) among animal species, the variations may be minimal. It is important to note that both 3T3-L1 and L6 cell lines have clone-specific traits and hence, fail to recapitulate the primary cells.

## Conclusion

In conclusion, the interaction between the skeletal muscle and adipose tissue is essential for the differential glucose uptake of the skeletal muscle cells under insulin-untreated or insulin-treated condition and this interaction, at least in part, is mediated *via* ROS production. Future studies need to clarify the role of this interaction in insulin signaling and glucose uptake.

## Availability of Data and Material

The datasets used and analyzed during the current study are available from the corresponding author on reasonable request.

## Author Contributions

AK has participated to the acquisition of all data, interpretation and the statistical analysis of data, and writing the paper. HS, HH, and TW have participated to analysis and interpretation of data. MS has participated to the interpretation and the statistical analysis of data, and writing the paper. All authors have read and approved the final manuscript.

## Conflict of Interest Statement

The authors declare that the research was conducted in the absence of any commercial or financial relationships that could be construed as a potential conflict of interest.

## Abbreviations

2-DOG, [^3^H]2-deoxyglucose; Glut, glucose transporter; ROS, reactive oxygen species; PPAR, peroxisome proliferator-activated receptor; PGC1α, PPARγ coactivator 1α; Ucp3, uncoupling protein 3; qRT-PCR, quantitative real-time reverse transcription polymerase chain reaction.
